# Neural correlates of conflict between gestures and words: A domain-specific role for a temporal-parietal complex

**DOI:** 10.1371/journal.pone.0173525

**Published:** 2017-03-09

**Authors:** J. Adam Noah, Swethasri Dravida, Xian Zhang, Shaul Yahil, Joy Hirsch

**Affiliations:** 1 Brain Function Laboratory, Department of Psychiatry, Yale School of Medicine, New Haven, Connecticut, United States of America; 2 Medical Scientist Training Program, Yale School of Medicine, New Haven, Connecticut, United States of America; 3 Department of Neurosciences, Washington University in St. Louis, St. Louis, Missouri, United States of America; 4 Department of Psychiatry, Yale School of Medicine, New Haven, Connecticut, United States of America; 5 Department of Neuroscience, Yale School of Medicine, New Haven, Connecticut, United States of America; 6 Department of Comparative Medicine, Yale School of Medicine, New Haven, Connecticut, United States of America; 7 Department of Medical Physics and Bioengineering, University College London, London, United Kingdom; Universiteit Gent, BELGIUM

## Abstract

The interpretation of social cues is a fundamental function of human social behavior, and resolution of inconsistencies between spoken and gestural cues plays an important role in successful interactions. To gain insight into these underlying neural processes, we compared neural responses in a traditional color/word conflict task and to a gesture/word conflict task to test hypotheses of domain-general and domain-specific conflict resolution. In the gesture task, recorded spoken words (“yes” and “no”) were presented simultaneously with video recordings of actors performing one of the following affirmative or negative gestures: thumbs up, thumbs down, head nodding (up and down), or head shaking (side-to-side), thereby generating congruent and incongruent communication stimuli between gesture and words. Participants identified the communicative intent of the gestures as either positive or negative. In the color task, participants were presented the words “red” and “green” in either red or green font and were asked to identify the color of the letters. We observed a classic “Stroop” behavioral interference effect, with participants showing increased response time for incongruent trials relative to congruent ones for both the gesture and color tasks. Hemodynamic signals acquired using functional near-infrared spectroscopy (fNIRS) were increased in the right dorsolateral prefrontal cortex (DLPFC) for incongruent trials relative to congruent trials for both tasks consistent with a common, domain-general mechanism for detecting conflict. However, activity in the left DLPFC and frontal eye fields and the right temporal-parietal junction (TPJ), superior temporal gyrus (STG), supramarginal gyrus (SMG), and primary and auditory association cortices was greater for the gesture task than the color task. Thus, in addition to domain-general conflict processing mechanisms, as suggested by common engagement of right DLPFC, socially specialized neural modules localized to the left DLPFC and right TPJ including adjacent homologous receptive language areas were engaged when processing conflicting communications. These findings contribute to an emerging view of specialization within the TPJ and adjacent areas for interpretation of social cues and indicate a role for the region in processing social conflict.

## Introduction

Spoken language is a gold standard for communication, but humans also rely on gestures as a fundamental source of social information [1]. Gestural elements in conversation are known to enhance verbal communication particularly when speakers agree or disagree, and interpretation of gesture may contradict verbal content [2]. Congruence between gestural and verbal communication has been associated with enhanced comprehension [3], whereas incongruence can serve as an alerting social cue. An incongruence between gestures and spoken language can signify that increased attention to the information stream is needed to parse meaning in a conversation. Interpretation of conflicting verbal and non-verbal cues is often considered an important part of lie detection. Frameworks for control in conflict tasks, such as the Stroop color task or the Wisconsin Card Sorting task, suggest activity in the dorsolateral prefrontal cortex (DLFPC) and anterior cingulate cortex (ACC) that provide a guided activation in top-down processing [4]. Other studies have added to this framework, indicating that prefrontal cortex structures contribute to domain-general processing of conflict while other regions of the brain display activity specific to the domain of the task, such as emotion or faces [5]. In this study, we aim to determine if spoken language when paired with incongruent body language, activates domain-general areas of cognitive processing as in the DLPFC and/or domain-specific areas including social and language areas of the cortex such as the temporal-parietal junction (TPJ) and adjacent homologues of receptive language processing areas, respectively.

The classic Stroop task [6, 7] introduced conflict between the written and perceptual domains of colors and words. Subsequent Stroop tasks have varied stimulus dimensions and/or response choices to investigate the neural correlates of conflict monitoring and resolution [8], emotional conflict [5], contextual and nonverbal components of social conflict [9], and integration of speech and iconic gestures [10]. Delays in reaction time to incongruent stimuli in these tasks are assumed to represent interference between conflicting stimulus dimensions, and associated activity in neural circuits localized to the prefrontal cortex (PFC) is usually taken as a marker of conflict processing in these tasks [4, 11].

The dorsolateral prefrontal cortex has been associated with general conflict detection and resolution [12, 13]. Together, the anterior cingulate cortex and the DLPFC are thought to form a network that detects conflict and recruits attention and response mechanisms in order to resolve the conflict in a task-relevant fashion [4, 11, 13]. For example, in a previous study, Zaki (2010) reported that reliance on nonverbal cues conveying facial and emotional information preferentially engaged areas such as the fusiform gyrus and amygdala, which are known to be involved in face and emotion processing. In another variant of the Stroop task, Egner and Hirsch (2005) found that conflict related to faces was resolved by up-regulation of task-specific processes and that the fusiform face area was more engaged when faces were the target, rather than the distracter, stimulus dimension. Crucially, both studies also found elevated DLPFC activity during incongruent trials [9, 12], furthering the hypothesis that this region is engaged in domain-general mechanisms of conflict processing alongside more domain-specific areas.

The temporal-parietal junction (TPJ) has been associated with social processing and consists of structures in the inferior parietal and posterior temporal lobes bilaterally [14–16]. The TPJ consists of nodes which are thought to play roles in theory of mind, intention analysis, and mentalizing, as well as coordination of gaze and processing of biological motion [15]. Overlap exists between the TPJ and components of a receptive language network, referred to as Wernicke’s area or its homologue [17], and previous electroencephalography (EEG) has shown that markers of integration between speech and iconic gestures may localize to parietal and midline sources [10]. Taken together, these findings suggest that the TPJ and receptive language sensitive systems would be strong candidates for domain-specific processing of conflict in various communication tasks. The overall goal of this study is to determine if conflict between body gestures and spoken language elicits domain-specific activity as expected for social and language receptive regions while also displaying traditional activity in domain-general areas of conflict monitoring in the DLPFC.

To address the question of specialization for conflict between spoken language and gestures, we measured regional neural activity using functional near-infrared spectroscopy (fNIRS). Functional NIRS is a technique that is well-suited for neuroimaging of tasks that cannot be easily performed in the confined space of an MRI or on individuals who are contraindicated for MRI because of the susceptibility effects of metal implants, dyskinesia, or anxiety orders, among other reasons. Although fNIRS has been used extensively in infant and child neuroimaging studies [18–23], the technique has not been widely applied to adult cognitive research.

The specific goals of this study involve identification of conflict detection systems of the brain that are used in evaluating congruency of social behaviors. We will determine how incongruency between spoken words and physical gestures influence neural activity. We specifically hypothesize that the TPJ and receptive language and homologous areas will respond more in tasks that involve social and language components than in the traditional color-word task. This hypothesis is based on the assumption that this complex will play a role in domain-specific conflict detection because of its role in social attention, language, memory, and social processing streams [15]. We also hypothesize increased prefrontal cortex activity will manifest in incongruent trials for both conflict tasks, indicating the general role of the prefrontal cortex in active maintenance of executive control and biasing of information to other parts of the brain needed to perform a task. Uncovering the neural underpinnings of social and communication conflict may ultimately inform models of psychiatric disorders, such as social anxiety [24], autism [25], and schizophrenia [26].

## Materials and methods

### Participants

Thirty-one healthy subjects (14 male, 17 female; mean age: 24.9 +/- 7.5 years; 100% right-handed [27]) participated in the experiment. Data were collected from two additional subjects that did not contribute to behavioral or neuroimaging results as their response times exceeded twice the mean of the group. Participants provided written informed consent in accordance with guidelines approved by the Yale University Human Investigation Committee (HIC #1501015178), which specifically approved this study. All data were obtained at the Yale School of Medicine, New Haven, Connecticut, USA.

### Stimuli

**Fig 1:**
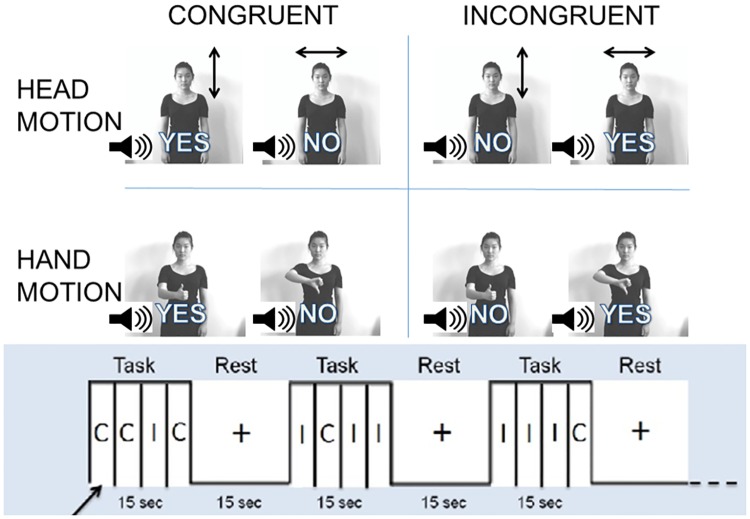
Gesture stroop stimuli and paradigm. A) Task design: Subjects indicated the meaning of the gesture as either positive or negative. Images represent video stills of four types of gesture: head nodding (up and down), head shaking (side-to-side), thumbs up, and thumbs down. Spoken words are super-imposed on video stills in each condition. Rows indicate the body part used in the gesture, i.e. head or hand. Columns indicate congruent and incongruent conditions, where gestures are congruent and incongruent with spoken words. B) Block design: 15s task block alternates with 15s rest block. 4 trials per block with ISI of 3.75s. Each block consisted predominantly of either congruent (C) or incongruent (I) trials, and contained one randomly positioned oddball trial.

Gesture tasks were generated by recording video and spoken language audio from four different actors: two male and two female. Actors in the videos were instructed to produce affirmative or negative gestures while maintaining neutral facial expressions. Audio was recorded in a separate session and multiplexed into the video sequences with the appropriate actor to coincide with the video onset using Adobe Premiere CS6 (San Jose, CA). Examples of congruent and incongruent gesture stimuli are presented in Fig 1A. The experimenter appearing in Fig 1A provided written consent to use her likeness in this manuscript. A Color Stroop task using the words “red” and “green” in congruent and incongruent colored letters was generated using a custom Python script implemented in PsychoPy [28].

### Paradigm

The gesture paradigm was developed to test whether incongruency between gestures and spoken language would elicit increased activity in the DLPFC and the TPJ and language-related areas. The main goal of this paradigm was to understand effects of conflicting communication cues. The decision-making paradigm consisted of evaluating four simple gestures: 1. Head nod (positive), 2. Head shake (negative), 3. Thumbs up (positive), and 4. Thumbs down (negative). The gestures were limited to positive and negative valance to simplify the social information and limit the amount of errors participants made when evaluating the task. During the task, a video of an actor performing one of the gestures was presented while the word “yes” or “no” was presented from an audio device. The voice saying the word was the same gender but otherwise independent of the actor seen in the video, who did not speak. Participants were told to indicate whether the meaning of the gesture in each video was affirmative or negative by pressing the right (affirmative) or left (negative) arrow key on a keyboard using the index and middle fingers of their right hands. Participants were given instructions to respond to the valance of the gesture but were also told that the audio may affect their ability to interpret the gesture. For the Color Stroop task a traditional two-color decision task was used to match the binary decision-making in the gesture task. During the task, participants indicated whether the color of the word was red (right arrow) or green (left arrow) by pressing the corresponding arrow key using the index and middle fingers of their right hands. For both tasks, participants were asked to respond as quickly and as accurately as possible to each trial.

Color and gesture stimuli were presented with an inter-stimulus interval (ISI) of 3.75s. For each task, twelve 15-second task blocks were interleaved with 15-second rest periods, during which participants focused on a fixation crosshair (Fig 1B). Prior Stroop studies have found reduction in reaction times due to repetition [7], so each task block contained one oddball trial to prevent repetition effects. Task blocks were of two kinds: congruent-dominant (3 congruent trials and 1 incongruent trial) and incongruent-dominant (3 incongruent trials and 1 congruent trial). The position of the oddball trial within each block was randomized and prevented the perception of a single-task block with repeated trials. For each event, the gender of the actor was randomized in the gesture task. Additionally, the type of gesture (head or hand) was also randomized. Each run contained 48 trials for a total run time of 6 minutes. All stimuli were presented with a custom Python script implemented in PsychoPy [28].

### Functional Near-Infrared Spectroscopy (fNIRS) signal acquisition

**Fig 2:**
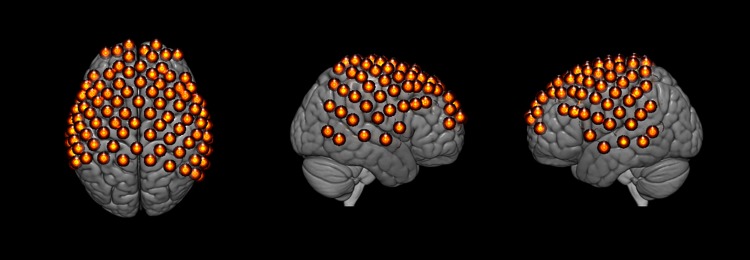
Functional near-infrared channel layout. Thirty emitter and twenty-nine detector pairs were placed at 3 cm intervals to generate a 98-channel layout covering frontal, parietal, and temporal areas as indicated by the orange spheres. Average channel locations are indicated in S1 Table.

Hemodynamic signals were acquired using a multichannel, continuous-wave fNIRS system (LABNIRS, Shimadzu Corp., Kyoto, Japan). Each participant was fitted with an optode cap with predefined channel distances of 3 cm. A lighted fiber-optic probe (Daiso, Hiroshima, Japan) was used to remove all hair from the optode channel prior to optode placement. Optodes consisting of 30 emitters and 29 detectors were arranged in a custom matrix, providing a total of 98 acquisition channels. The specific layout with the coverage of the optode channels is shown in Fig 2 and the mean channel coordinates and locations are detailed in S1 Table. Placement of the most anterior channel of the optode holder cap was centered 1 cm above nasion. To assure acceptable signal-to-noise ratios, resistance was measured for each channel prior to recording, and adjustments were made for each optode until all channels met the minimum criteria defined in the LABNIRS recording software [29–31].

As is standard for Shimadzu NIRS devices, each emitter fiber connects to laser diodes at three wavelengths (780nm, 805nm, 830nm). Raw optical density variations were translated into changes in relative chromophore concentrations using a modified Beer-Lambert equation, as described previously [32–34]. Signals were recorded at 27msec per sample.

### Optode localization

The anatomical locations of optodes in relation to standard head landmarks including inion, nasion, Cz, and left (T3) and right (T4) ears, were determined for each participant using a Patriot 3D Digitizer (Polhemus, Colchester, VT) and linear transform techniques as previously described [35–39]. MNI coordinates for the channels were obtained using the NIRS_SPM software [40] with MATLAB (Mathworks, Natick, MA), and the corresponding anatomical locations of each channel were determined by the atlas provided [41, 42] and shown in S1 Table.

### Data analysis

#### Reaction time

For color trials, reaction time was calculated as the difference in time from the onset of the stimulus to the time of the button press. In the case of the gesture trials, four independent raters determined the time it took to resolve the meaning of the gesture displayed in every video. The median of these inter-rater values for each video was taken as the gesture stimulus onset time. This was used to produce a reaction time measured from the stimulus onset time in the video to the time of a button press. One-tailed paired t-tests were used to determine differences in reaction times.

#### fNIRS signal processing

Both oxyhemoglobin (OxyHb) and deoxyhemoglobin (deOxyHb) fNIRS signals have been shown to correspond to blood oxygen level-dependent (BOLD) responses measured by functional magnetic resonance imaging (fMRI) [43–48]. However, the deoxyhemoglobin signal acquired by fNIRS is less susceptible to systemic artifacts [48, 49]. Due to the increased functional specificity it is reported in this study although the deOxyHb is typically smaller than the OxyHb signal. After conversion from optical density to deOxyHb concentrations, signals were detrended with the root mean square of the residual left over after deconvolution with a standard hemodynamic response function (HRF) and low-pass filtered at 0.1 Hz per channel [50]. Baseline drift was modeled by detrending using a wavelet detrending [40]. Channels without signals were identified automatically and removed from the analysis if the root mean square of the raw data trace was more than 10 times the average signal for each individual subject.

Systemic effects, such as blood pressure, respiration, and blood flow variation have been previously shown to alter relative blood hemoglobin concentrations [49, 51]. These global components were removed using a PCA-spatial filter [52] prior to general linear model (GLM) analysis [50]. Functional NIRS data were down-sampled 10-fold for an effective sample rate of 0.9 sec. The 98-channel fNIRS data were reshaped into 4x4x4x133 images for the first-level GLM analysis using SPM8 [53].

#### Contrast effects

Comparisons between conditions were based on the general linear model for fNIRS [53]. Event epochs were convolved with a standard HRF, which was then fitted to the data, providing individual beta values for each participant across incongruent and congruent conditions. Images were rendered on a standardized MNI brain template using MRIcroGL (http://www.mccauslandcenter.sc.edu/mricrogl/home/). Monte Carlo simulations were performed using 30 data sets. For each permutation, beta values for randomly chosen subjects were multiplied by -1, where the expected group result would be no activity. Any positive results would be considered false. This simulation was repeated 1000 times to determine false positive rates. The results of this simulation with multiple corrected and uncorrected p value thresholds with corresponding cluster thresholds are shown in Table 1. Table values represent the percent of false positive cases. In particular, a cluster size of 70 and an uncorrected p-value of 0.001 results in a value corrected for multiple comparisons of 0.0495, shown in bold in Table 1.

**Table 1:** Cluster simulation results.

		Uncorrected P-value			
		0.05	0.01	0.001	0.0001
Cluster Size	10	1	0.9194	0.2187	0.0205
	30	1	0.7753	0.1086	0.007
	50	1	0.6697	0.0691	0.004
	70	0.9965	0.5881	**0.0495**	0.002

#### Channel comparisons

To further reduce the probability of false positives due to multiple voxel-wise comparisons, we also analyzed the data using a channel-wise approach. While this approach is not independent of the voxel-wise SPM technique, it does reduce the number of comparisons to 98 channels. Each participant’s channel locations were converted to MNI space, and individual subject data was registered to the median channel coordinate using a non-linear interpolation method similar to methods described in [52]. Once in normalized space, channel-wise comparisons were used across conditions. Results that are reported as significant had to reach two criteria. First, results were required to reach an uncorrected threshold of p<0.001, with a cluster threshold of 70, resulting in a corrected threshold of p < 0.05 for voxel-wise comparisons. A second criterion required any result to also reach p < 0.05 for channel-wise comparisons in corresponding channels. Any result that did not meet the voxel-wise criterion is referred to as “active” rather than significant.

## Results

### Behavior

Reaction times for both gesture and color tasks increased for the incongruent trials. In the case of the Gesture Stroop task, group mean reaction times for incongruent trials were 1405 ± 171ms (Standard Error of Measurement, SEM) relative to congruent (1365 ± 169ms) (one-tailed paired t-test, t = 3.667, p = 0.001, df: 30). In the case of the Color Stroop, mean reaction times for incongruent trials were 764 ± 191ms relative to congruent (658 ± 153ms) (one-tailed paired t-test, t = 8.011, p = 6.1x10^-9^, df: 30). The group mean reaction time for all gesture trials (1385 ± 167ms) was significantly greater than that of the color trials (711 ± 169ms) (one-tailed paired t-test, t = 30.318, p = 4.6x10^-24^, df: 30). The group mean reaction time for all incongruent trials (1085 ± 166ms) was significantly greater than that of the congruent trials (1012 ± 150ms) one-tailed paired t-test, t = 9.145, p = 3.5x10^-10^, df: 30). There was no difference between the number of incorrect responses for gesture and color tasks (98.2% correct for the gesture task and 98.7% for the color task). All behavioral analyses were performed in MATLAB R2014A, version 8.3.0.532 (The MathWorks Inc., Natick, MA). We counterbalanced the binary nature of the “yes” and “no” responses in the gesture task with a two-color Stroop task. The two-color task did result in similar accuracy results to the gesture task, but may have been easier for subjects and thus may have contributed to the shorter response times seen in the color Stroop task.

### Hemodynamic imaging results

#### Contrast results: Incongruent vs congruent trials

**Fig 3:**
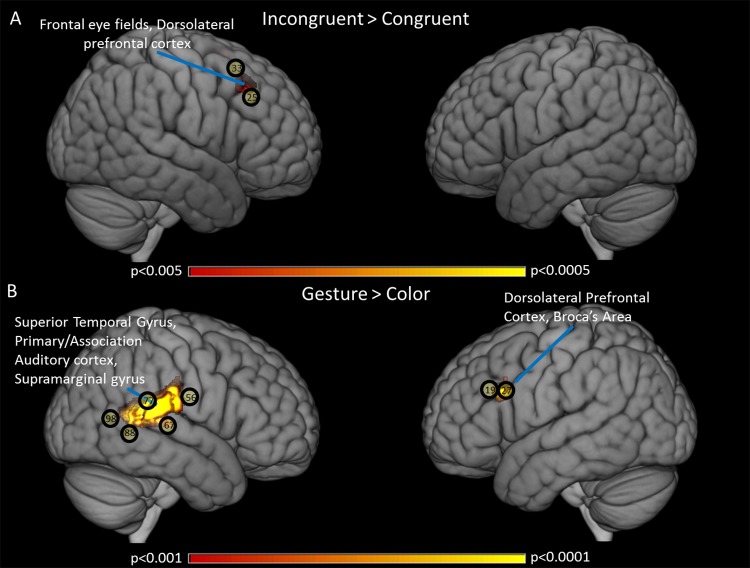
Contrast effects: deOxyHb signals, n = 31. A) Activated clusters indicate the domain-general results of the Incongruent > Congruent contrast (p<0.005), with activity present in right DLPFC. B) Activated clusters indicate the domain-specific results of the Gesture > Color contrast (p<0.001), with activity present in right STG and left DLPFC. Black circles indicate the channel number and location of the significant channels (p<0.05) from the channel-wise analysis.

To determine the domain-general effect of congruency, we analyzed the contrast of incongruent trials > congruent trials, combined across both the gesture and color tasks. We report brain areas showing positive activity for both SPM analysis and channel-wise analysis. An overview of the SPM results is presented in Fig 3, Table 2. Fig 3A shows renderings representing Incongruent > Congruent trials and the mean locations of the activated channels. The cluster of activity is shown in the right DLPFC with peak voxel at (36, 26, 46) (p≤0.005 (uncorrected), t = 3.26, n of voxels = 123). These results were supported by the channel-wise analysis (Table 3), indicated on the figure by open circles including channel numbers, which showed two active adjacent channels in the right DLPFC: channel 25 (t = 1.91, p = 0.033) and channel 33 (t = 1.81, p = 0.04).

**Table 2:** Contrast results from voxel-wise analysis (deOxyHb signals).

Contrast	Contrast Threshold	Peak MNI-coordinates	T	P	# Voxels	BA	Anatomical Area	Probability
Incongruent > Congruent	p<0.005	{36, 26, 46}	3.26	0.0014	123	8	Frontal eye fields	0.89
						9	Dorsolateral prefrontal cortex	0.11
Gesture > Color	p<0.001	{70, -32, 14}	6.05	6.1x10^-7^	795	22	Superior Temporal Gyrus	0.47
						42	Primary and Auditory Association Cortex	0.26
						40	Supramarginal gyrus part of Wernicke's area	0.24
		{-54, 24, 28}	5.10	8.8x10^-6^	120	9	Dorsolateral prefrontal cortex	0.42
						46	Dorsolateral prefrontal cortex	0.31
						45	Pars triangularis Broca's area	0.22

Peak MNI coordinates, T-values, and p values for each contrast are listed. Colors indicate separate clusters. Coordinates are based on the MNI system and (-) on the x-axis indicates left hemisphere. MNI coordinates were converted to Talairach coordinates to generate cluster labels. BA = Brodmann’s Area.

**Table 3:** Contrast results from channel-wise analysis (deOxyHb signals).

Contrast (p<0.05)	Channel #	Average MNI coordinates		T	p	BA	Anatomical Area	Probability
Incongruent > Congruent	25	{54.5, 26, 33.7}		1.91	0.033	9	Dorsolateral prefrontal cortex	0.65
						46	Dorsolateral prefrontal cortex	0.33
						45	pars triangularis Broca's area	0.02
	33	{47.8, 17.6, 51.3}		1.81	0.040	8	Includes Frontal eye fields	0.76
						6	Pre Motor and Supplementary Motor Cortex	0.24
Gesture > Color	56	{69.9, -11.1, 19.2}		3.10	0.002	43	Subcentral area	0.45
						42	Primary and Auditory Association Cortex	0.12
						6	Pre Motor and Supplementary Motor Cortex	0.09
						22	Superior Temporal Gyrus	0.08
						3	Primary Somatosensory Cortex	0.07
						4	Primary Motor Cortex	0.07
						1	Primary Somatosensory Cortex	0.06
						40	Supramarginal gyrus part of Wernicke's area	0.05
						2	Primary Somatosensory Cortex	0.00
	67	{72.4, -25.8, 2.8}		3.77	0.0003	22	Superior Temporal Gyrus	0.48
						21	Middle Temporal gyrus	0.26
						42	Primary and Auditory Association Cortex	0.26
	77	{70.6, -38.1, 17}		2.36	0.012	22	Superior Temporal Gyrus	0.65
						40	Supramarginal gyrus part of Wernicke's area	0.19
						42	Primary and Auditory Association Cortex	0.15
	88	{67.4, -51.4, -2.1}		3.54	0.001	21	Middle Temporal gyrus	0.62
						37	Fusiform gyrus	0.31
						22	Superior Temporal Gyrus	0.07
	98	{61.4, -63.7, 6.8}		3.34	0.001	37	Fusiform gyrus	0.30
						21	Middle Temporal gyrus	0.27
						39	Angular gyrus, part of Wernicke's area	0.19
						19	V3	0.13
						22	Superior Temporal Gyrus	0.12
	19	{-53.1, 27.9, 28.1}		2.02	0.026	46	Dorsolateral prefrontal cortex	0.56
						9	Dorsolateral prefrontal cortex	0.26
						45	pars triangularis Broca's area	0.17
	27	{-58.0, 17.3, 26.4}		4.64	0.00004	9	Dorsolateral prefrontal cortex	0.50
						45	pars triangularis Broca's area	0.31
						44	pars opercularis, part of Broca's area	0.12
						46	Dorsolateral prefrontal cortex	0.06

Colors indicate separate clusters. Coordinates are based on the MNI system and (-) on the x-axis indicates left hemisphere. MNI coordinates were converted to Talairach coordinates to generate cluster labels. BA = Brodmann’s Area.

#### Contrast results: Gesture vs color tasks

**Fig 4:**
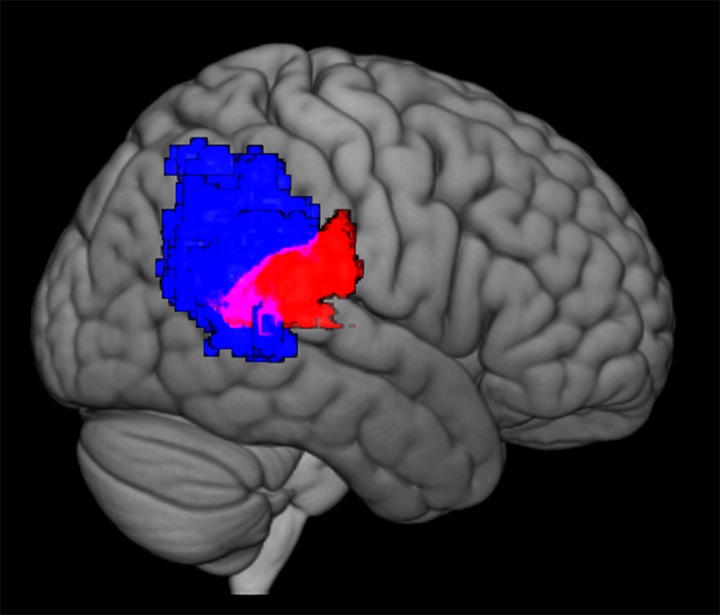
Overlap of neurosynth right TPJ and gesture > color activity. Red area represents left temporal-parietal region of activity from the Gesture > Color contrast, p<0.001. Blue area shows forward inference map of the rTPJ from Neurosynth (http://neurosynth.org) meta-analysis of 92 studies. Black dotted line surrounds area of overlap.

To investigate the effects of gesture, we compared the gesture task > color task, combining incongruent and congruent trials for voxel-wise activity and the corresponding channel-wise activity (Table 3) as seen in Fig 4B. A significant cluster is seen in the left DLPFC (-54, 24, 28) (p≤0.05 (corrected), t = 5.10, n of voxels = 120). The channel-wise analysis showed two active channels in this area: channel 19 (t = 2.02, p = 0.026) and channel 27 (t = 4.64, p = 0.00003). The right hemisphere cluster is located in the superior temporal gyrus, primary auditory cortex, and supramarginal gyrus with peak voxel at (70, -32, 14) (p<0.05 (corrected), t = 6.05, n of voxels = 795) and corresponded with activity in five channels: channel 56 (t = 3.10, p = 0.002), channel 67 (t = 3.77, p = 0.0004), channel 77 (t = 2.36, p = 0.012), channel 88 (t = 3.54, p = 0.001), and channel 98 (t = 3.34, p = 0.001).

Fig 4 shows the overlap between the posterior cluster in the right hemisphere contrast (red) and the forward inference map of the rTPJ from a Neurosynth meta-analysis of 92 studies [54]. Neurosynth is an online meta-analysis tool that uses references to specific terms to generate maps of activity based on the combined findings of many published studies. In this case, the term “TPJ” was found in 92 studies with 3460 activations referenced. A statistical analysis is performed on studies that do and do not reference the TPJ, and a statistical inference map of the TPJ is generated using the coordinates reported in these studies. We calculated that 23% of the total active voxels in this cluster were located within the Neurosynth inference map of the TPJ. The remaining areas include language processing areas such as the STG, SMG and associated auditory systems.

## Discussion

The findings of this study identify socially sensitive neural circuitry using a novel gestural Stroop task. Neuroimaging with fNIRS revealed that conflicts between both gestures and words elicited activity reflecting overlapping domain-general processing in the right DLPFC, which supports the function of this area as a domain-general hub for conflict-related tasks. The gesture task activated the right TPJ and receptive language areas to a larger extent than the color task These findings extend the known general functions of the TPJ to suggest a specific role in the detection and processing of social conflict. Prior neuroimaging results are consistent with the hypothesis that conflict between gestures and spoken words engage domain-general areas of the brain with respect to conflict control and decision-making [9, 11–13] including the DLPFC. We found that activity in the temporal-parietal junction, thought to be associated with various aspects of social cognition [15], was also increased in conflict-related trials that were specific to communication.

### Prefrontal cortex and domain-general conflict processing

Activity observed in the frontal cortex, including the DLPFC, is known to function in domain-general features of conflict processing, and particularly in the implementation of task-specific responses [11–13]. Activity in the DLPFC is commonly associated with studies of conflict detection and resolution [13], and is thought to bias motor and pre-motor cortices toward selecting the contextually correct or task-evoked interpretation of the stimulus [9, 11–13]. Although fNIRS signals are limited to superficial cortical structures with minimal sensitivity to deeper structures, the common activity from DLPFC observed in both gesture and color tasks along with the observed behavioral interference effects suggests the involvement of a system for conflict processing that is similar to those previously described in the fMRI literature [5, 8, 9].

A number of decision-making tasks have shown right lateralized prefrontal cortical activity [55–57] consistent with our findings of right-lateralized frontal eye fields/DLPFC in response to all incongruent > congruent trials combined across task. However, we also report that activity in the left DLPFC was greater when participants responded to gesture videos than when they responded to color words. We speculate that right hemisphere DLPFC may operate to bias participant responses toward interpretation of the gesture (as participants were directed), and is more highly engaged during tasks in which interpretation of the stimuli, and therefore resolution of the conflict, is more difficult. Left hemisphere DLPFC may be upregulated by domain-specific demands of the language and social aspects of the task.

### Temporal-parietal junction and social language processing

Domain-specific activity in the gesture task is consistent with meta-analytical demarcations of the TPJ and its proposed roles in social cognition, including the processing of language and biological motion [15, 16]. Other evidence for the role of the TPJ and social phenomenon include EEG signatures for gesture-word integration within central and parietal sources [10]. Additionally, to the supramarginal gyrus has also been implicated in gestural comprehension [58]. The present findings contribute a novel neural complex including the TPJ and the receptive language homologues substrate that resolve contradictory interpersonal communications.

Understanding the functional specifications of this complex is relevant to psychiatric disorders. For example, hyperactivation of the TPJ has been observed in people with schizophrenia, and may be related to the hallucinatory sense of action and agency [26]. Elevations in TPJ activity have also been detected during inwardly focused attention during social situations in people with high levels of social anxiety [24].

### Limitations

The findings of this study suggest that activity in the TPJ specific to the gesture task is related to domain-specific conflict processing. However, it is also possible that aspects of the stimuli other than the social nature of the gestures contributed to these responses. Here, we show increased activity in the TPJ/STG area for all gesture trials > color trials. More closely matched task paradigms may be able to add granularity to our understanding of the difference between social communication and cognitive processing. For example, using the same stimuli with two tasks could accomplish this goal. While many studies have shown high correlation between the BOLD signal recorded using fMRI and the oxy- and deoxy-hemoglobin signals recorded with fNIRS [44, 45], a number of systemic artifacts have been shown to be prevalent in the oxy-hemoglobin signal [59–61]. Because of these systemic artifacts, we used the deoxyhemoglobin signal for functional neuroimaging analysis here. While the deoxyhemoglobin signal is less susceptible to global or systemic artifacts, it is smaller and has a lower signal to noise ratio than the oxyhemoglobin signal resulting in a reduced signal-to-noise ratio, but greater sensitivity to neutrally-mediated effects [62, 63]. Future studies may benefit by increasing signal strength with increased repetitions of the task.

While both clusters of activity in the TPJ in the Gesture > Color contrast seen in Fig 3B are thresholded at p < 0.05 (corrected), activity in the Incongruent < Congruent contrast does not meet cluster correction criteria and thus, future repetition of the congruency results is recommended. While we have performed Monte Carlo simulations to determine corrected thresholds based on cluster size, we acknowledge the limits of cluster based thresholding as recently outlined by Eklund, et al. [64]. To further address spatial correlation in cluster correction, we also have reduced the number of voxel-wise comparisons using a channel-wise analysis and we report results in both voxel-wise and channel-wise analyses in the results.

Functional NIRS records information from superficial cortical areas, including the DLPFC and the TPJ, but not brain areas that are medial or deeper than two-three centimeters in the parenchyma [44], including the cingulate cortex and basal ganglia. Because of this, comparison of previous fMRI results from the Color Stroop task to those conducted using fNIRS imaging methods is limited. Further, in this study we were not able to determine if the visual cortex was active in a domain-specific fashion for the color Stroop task as we did not have optodes covering the occipital lobe.

We used video representations of humans performing the gesture tasks instead of live confederate performers. While we did this to assure all participants received a similar set of stimuli, the benefits of using fNIRS to record brain function in ecologically valid tasks were not optimally utilized. Future studies will be performed in which pairs or groups of individuals will perform similar gesturally conflicting tasks to further understand the role of the TPJ and language areas in domain-specific conflict processing in natural human interaction.

## Supporting information

S1 Table Channels, group-averaged coordinates, anatomical regions, and atlas-based probabilities. (PDF)
