# Supplementary material for: Neural correlates of conflict between gestures and words: A domain-specific role for a temporal-parietal complex
Source: PLoS One. 2017 Mar 9;12(3):e0173525. doi: 10.1371/journal.pone.0173525 (PMC5344449; doi:10.1371/journal.pone.0173525)
Supplement: S1 Table — (PDF) [file pone.0173525.s001.pdf]

**S1 Table. Channels, group-averaged coordinates, anatomical regions, and atlas-based probabilities.**

| Channel number | MNI Coordinates* X | Y | Z | BA** | Anatomical Region | Probability |
|---|---|---|---|---|---|---|
| 1 | -34.1 | 62.8 | 9.6 | 10 | Frontopolar area | 1.00 |
| 2 | -10.6 | 70.0 | 18.8 | 10 | Frontopolar area | 1.00 |
| 3 | 18.7 | 69.2 | 20.0 | 10 | Frontopolar area | 1.00 |
| 4 | 40.1 | 59.6 | 14.0 | 10 | Frontopolar area | 0.96 |
| | | | | 46 | Dorsolateral prefrontal cortex | 0.04 |
| 5 | -21.6 | 63.4 | 26.8 | 9 | Dorsolateral prefrontal cortex | 0.11 |
| | | | | 10 | Frontopolar area | 0.89 |
| 6 | 5.3 | 63.1 | 32.2 | 9 | Dorsolateral prefrontal cortex | 0.30 |
| | | | | 10 | Frontopolar area | 0.70 |
| 7 | 27.6 | 60.6 | 28.4 | 9 | Dorsolateral prefrontal cortex | 0.24 |
| | | | | 10 | Frontopolar area | 0.76 |
| 8 | -10.1 | 58.1 | 40.8 | 8 | Includes Frontal eye fields | 0.09 |
| | | | | 9 | Dorsolateral prefrontal cortex | 0.85 |
| | | | | 10 | Frontopolar area | 0.06 |
| 9 | 14.8 | 57.0 | 40.8 | 8 | Includes Frontal eye fields | 0.12 |
| | | | | 9 | Dorsolateral prefrontal cortex | 0.86 |
| | | | | 10 | Frontopolar area | 0.02 |
| 10 | -19.6 | 48.7 | 45.5 | 8 | Includes Frontal eye fields | 0.59 |
| | | | | 9 | Dorsolateral prefrontal cortex | 0.41 |

| | | | | | | |
|---|---|---|---|---|---|---|
| 11 | 2.5 | 48.5 | 49.1 | 8 | Includes Frontal eye fields | 0.83 |
| | | | | 9 | Dorsolateral prefrontal cortex | 0.17 |
| 12 | 23.5 | 46.9 | 47.1 | 8 | Includes Frontal eye fields | 0.73 |
| | | | | 9 | Dorsolateral prefrontal cortex | 0.27 |
| 13 | -46.5 | 38.7 | 29.8 | 9 | Dorsolateral prefrontal cortex | 0.18 |
| | | | | 10 | Frontopolar area | 0.02 |
| | | | | 46 | Dorsolateral prefrontal cortex | 0.80 |
| 14 | -30.4 | 39.3 | 46.6 | 8 | Includes Frontal eye fields | 0.75 |
| | | | | 9 | Dorsolateral prefrontal cortex | 0.25 |
| 15 | -10.9 | 41.1 | 55.7 | 6 | Pre Motor and Supplementary Motor Cortex | 0.05 |
| | | | | 8 | Includes Frontal eye fields | 0.95 |
| 16 | 13.1 | 40.7 | 56.4 | 6 | Pre Motor and Supplementary Motor Cortex | 0.08 |
| | | | | 8 | Includes Frontal eye fields | 0.92 |
| 17 | 32.2 | 37.3 | 49.4 | 8 | Includes Frontal eye fields | 0.92 |
| | | | | 9 | Dorsolateral prefrontal cortex | 0.08 |
| 18 | 48.4 | 35.8 | 35.1 | 9 | Dorsolateral prefrontal cortex | 0.56 |
| | | | | 46 | Dorsolateral prefrontal cortex | 0.44 |
| 19 | -53.1 | 27.9 | 28.1 | 9 | Dorsolateral prefrontal cortex | 0.26 |
| | | | | 45 | pars triangularis Broca's area | 0.17 |
| | | | | 46 | Dorsolateral prefrontal cortex | 0.56 |
| 20 | -40.5 | 28.9 | 47.4 | 8 | Includes Frontal eye fields | 0.82 |
| | | | | 9 | Dorsolateral prefrontal cortex | 0.18 |
| 21 | -20.1 | 30.5 | 59.8 | 6 | Pre Motor and Supplementary Motor Cortex | 0.43 |
| | | | | 8 | Includes Frontal eye fields | 0.57 |

| | | | | | | |
|---|---|---|---|---|---|---|
| 22 | 1.2 | 31.4 | 60.3 | 6 | Pre Motor and Supplementary Motor Cortex | 0.52 |
| | | | | 8 | Includes Frontal eye fields | 0.48 |
| 23 | 21.9 | 29.6 | 60.4 | 6 | Pre Motor and Supplementary Motor Cortex | 0.51 |
| | | | | 8 | Includes Frontal eye fields | 0.49 |
| 24 | 41.9 | 26.5 | 51.2 | 8 | Includes Frontal eye fields | 0.99 |
| | | | | 9 | Dorsolateral prefrontal cortex | 0.01 |
| 25 | 54.5 | 26.0 | 33.7 | 9 | Dorsolateral prefrontal cortex | 0.65 |
| | | | | 45 | pars triangularis Broca's area | 0.02 |
| | | | | 46 | Dorsolateral prefrontal cortex | 0.33 |
| 26 | -60.3 | 12.7 | 3.6 | 6 | Pre Motor and Supplementary Motor Cortex | 0.05 |
| | | | | 22 | Superior Temporal Gyrus | 0.42 |
| | | | | 38 | Temporopolar area | 0.00 |
| | | | | 44 | pars opercularis, part of Broca's area | 0.29 |
| | | | | 45 | pars triangularis Broca's area | 0.15 |
| | | | | 47 | Inferior prefrontal gyrus | 0.08 |
| 27 | -58.0 | 17.3 | 26.4 | 9 | Dorsolateral prefrontal cortex | 0.50 |
| | | | | 44 | pars opercularis, part of Broca's area | 0.12 |
| | | | | 45 | pars triangularis Broca's area | 0.31 |
| | | | | 46 | Dorsolateral prefrontal cortex | 0.06 |
| 28 | -47.5 | 20.4 | 46.7 | 6 | Pre Motor and Supplementary Motor Cortex | 0.04 |
| | | | | 8 | Includes Frontal eye fields | 0.73 |
| | | | | 9 | Dorsolateral prefrontal cortex | 0.23 |
| 29 | -32.3 | 20.8 | 60.1 | 6 | Pre Motor and Supplementary Motor Cortex | 0.43 |
| | | | | 8 | Includes Frontal eye fields | 0.57 |
| 30 | -12.7 | 22.3 | 67.0 | 6 | Pre Motor and Supplementary Motor Cortex | 1.00 |
| 31 | 13.3 | 22.0 | 67.0 | 6 | Pre Motor and Supplementary Motor Cortex | 1.00 |

| | | | | | | |
|---|---|---|---|---|---|---|
| 32 | 31.7 | 19.5 | 62.1 | 6 | Pre Motor and Supplementary Motor Cortex | 0.67 |
| | | | | 8 | Includes Frontal eye fields | 0.33 |
| 33 | 47.8 | 17.6 | 51.3 | 6 | Pre Motor and Supplementary Motor Cortex | 0.24 |
| | | | | 8 | Includes Frontal eye fields | 0.76 |
| 34 | 60.6 | 13.0 | 32.3 | 6 | Pre Motor and Supplementary Motor Cortex | 0.09 |
| | | | | 9 | Dorsolateral prefrontal cortex | 0.84 |
| | | | | 45 | pars triangularis Broca's area | 0.07 |
| 35 | 63.9 | 11.0 | 10.6 | 6 | Pre Motor and Supplementary Motor Cortex | 0.21 |
| | | | | 22 | Superior Temporal Gyrus | 0.13 |
| | | | | 44 | pars opercularis, part of Broca's area | 0.54 |
| | | | | 45 | pars triangularis Broca's area | 0.12 |
| 36 | -65.8 | -3.0 | -8.0 | 21 | Middle Temporal gyrus | 0.90 |
| | | | | 22 | Superior Temporal Gyrus | 0.10 |
| 37 | -63.8 | 3.7 | 22.4 | 4 | Primary Motor Cortex | 0.03 |
| | | | | 6 | Pre Motor and Supplementary Motor Cortex | 0.62 |
| | | | | 9 | Dorsolateral prefrontal cortex | 0.14 |
| | | | | 43 | Subcentral area | 0.02 |
| | | | | 44 | pars opercularis, part of Broca's area | 0.13 |
| | | | | 45 | pars triangularis Broca's area | 0.07 |
| 38 | -55.7 | 7.0 | 41.9 | 6 | Pre Motor and Supplementary Motor Cortex | 0.53 |
| | | | | 8 | Includes Frontal eye fields | 0.16 |
| | | | | 9 | Dorsolateral prefrontal cortex | 0.31 |
| 39 | -41.6 | 12.4 | 58.6 | 6 | Pre Motor and Supplementary Motor Cortex | 0.68 |
| | | | | 8 | Includes Frontal eye fields | 0.32 |
| 40 | -22.1 | 12.4 | 68.8 | 6 | Pre Motor and Supplementary Motor Cortex | 1.00 |
| 41 | -0.9 | 11.0 | 70.4 | 6 | Pre Motor and Supplementary Motor Cortex | 1.00 |
| 42 | 21.3 | 10.1 | 70.7 | 6 | Pre Motor and Supplementary Motor Cortex | 1.00 |

| | | | | | | |
|---|---|---|---|---|---|---|
| 43 | 41.5 | 8.1 | 61.2 | 6 | Pre Motor and Supplementary Motor Cortex | 0.91 |
| | | | | 8 | Includes Frontal eye fields | 0.09 |
| 44 | 57.5 | 3.0 | 47.1 | 6 | Pre Motor and Supplementary Motor Cortex | 0.85 |
| | | | | 8 | Includes Frontal eye fields | 0.10 |
| | | | | 9 | Dorsolateral prefrontal cortex | 0.06 |
| 45 | 66.7 | 0.7 | 27.6 | 4 | Primary Motor Cortex | 0.05 |
| | | | | 6 | Pre Motor and Supplementary Motor Cortex | 0.77 |
| | | | | 9 | Dorsolateral prefrontal cortex | 0.16 |
| | | | | 43 | Subcentral area | 0.01 |
| | | | | 44 | pars opercularis, part of Broca's area | 0.01 |
| | | | | 45 | pars triangularis Broca's area | 0.01 |
| 46 | 68.3 | -3.9 | -1.9 | 21 | Middle Temporal gyrus | 0.59 |
| | | | | 22 | Superior Temporal Gyrus | 0.40 |
| | | | | 42 | Primary and Auditory Association Cortex | 0.01 |
| 47 | -67.1 | -8.2 | 15.3 | 4 | Primary Motor Cortex | 0.04 |
| | | | | 6 | Pre Motor and Supplementary Motor Cortex | 0.14 |
| | | | | 22 | Superior Temporal Gyrus | 0.23 |
| | | | | 42 | Primary and Auditory Association Cortex | 0.19 |
| | | | | 43 | Subcentral area | 0.39 |
| 48 | -62.6 | -5.0 | 36.9 | 4 | Primary Motor Cortex | 0.04 |
| | | | | 6 | Pre Motor and Supplementary Motor Cortex | 0.96 |
| 49 | -50.1 | -0.5 | 54.8 | 3 | Primary Somatosensory Cortex | 0.00 |
| | | | | 4 | Primary Motor Cortex | 0.07 |
| | | | | 6 | Pre Motor and Supplementary Motor Cortex | 0.92 |
| 50 | -32.9 | 2.0 | 66.7 | 6 | Pre Motor and Supplementary Motor Cortex | 1.00 |
| 51 | -13.5 | 0.6 | 74.4 | 6 | Pre Motor and Supplementary Motor Cortex | 1.00 |
| 52 | 12.9 | 0.0 | 74.9 | 6 | Pre Motor and Supplementary Motor Cortex | 1.00 |
| 53 | 30.7 | -3.3 | 69.5 | 6 | Pre Motor and Supplementary Motor Cortex | 1.00 |
| 54 | 50.0 | -6.6 | 58.9 | 3 | Primary Somatosensory Cortex | 0.12 |
| | | | | 4 | Primary Motor Cortex | 0.18 |
| | | | | 6 | Pre Motor and Supplementary Motor Cortex | 0.70 |
| 55 | 63.8 | -9.1 | 42.1 | 3 | Primary Somatosensory Cortex | 0.06 |
| | | | | 4 | Primary Motor Cortex | 0.07 |

| | | | | | | |
|---|---|---|---|---|---|---|
| | | | | 6 | Pre Motor and Supplementary Motor Cortex | 0.87 |
| 56 | 69.9 | -11.1 | 19.2 | 1 | Primary Somatosensory Cortex | 0.06 |
| | | | | 2 | Primary Somatosensory Cortex | 0.00 |
| | | | | 3 | Primary Somatosensory Cortex | 0.07 |
| | | | | 4 | Primary Motor Cortex | 0.07 |
| | | | | 6 | Pre Motor and Supplementary Motor Cortex | 0.09 |
| | | | | 22 | Superior Temporal Gyrus | 0.08 |
| | | | | 40 | Supramarginal gyrus part of Wernicke's area | 0.05 |
| | | | | 42 | Primary and Auditory Association Cortex | 0.12 |
| | | | | 43 | Subcentral area | 0.45 |
| 57 | -70.0 | -21.6 | -1.0 | 21 | Middle Temporal gyrus | 0.57 |
| | | | | 22 | Superior Temporal Gyrus | 0.30 |
| | | | | 42 | Primary and Auditory Association Cortex | 0.13 |
| 58 | -67.1 | -17.1 | 28.5 | 1 | Primary Somatosensory Cortex | 0.18 |
| | | | | 2 | Primary Somatosensory Cortex | 0.23 |
| | | | | 3 | Primary Somatosensory Cortex | 0.13 |
| | | | | 4 | Primary Motor Cortex | 0.06 |
| | | | | 6 | Pre Motor and Supplementary Motor Cortex | 0.11 |
| | | | | 40 | Supramarginal gyrus part of Wernicke's area | 0.10 |
| | | | | 43 | Subcentral area | 0.19 |
| 59 | -59.3 | -15.0 | 48.3 | 1 | Primary Somatosensory Cortex | 0.10 |
| | | | | 2 | Primary Somatosensory Cortex | 0.09 |
| | | | | 3 | Primary Somatosensory Cortex | 0.22 |
| | | | | 4 | Primary Motor Cortex | 0.14 |
| | | | | 6 | Pre Motor and Supplementary Motor Cortex | 0.44 |
| 60 | -43.5 | -10.9 | 63.8 | 3 | Primary Somatosensory Cortex | 0.12 |
| | | | | 4 | Primary Motor Cortex | 0.13 |
| | | | | 6 | Pre Motor and Supplementary Motor Cortex | 0.75 |
| 61 | -23.4 | -10.6 | 74.7 | 6 | Pre Motor and Supplementary Motor Cortex | 1.00 |
| 62 | -3.2 | -9.9 | 75.8 | 6 | Pre Motor and Supplementary Motor Cortex | 1.00 |
| 63 | 21.2 | -11.9 | 75.7 | 6 | Pre Motor and Supplementary Motor Cortex | 1.00 |
| 64 | 42.7 | -16.8 | 68.0 | 3 | Primary Somatosensory Cortex | 0.20 |

| | | | | | | |
|---|---|---|---|---|---|---|
| | | | | 4 | Primary Motor Cortex | 0.17 |
| | | | | 6 | Pre Motor and Supplementary Motor Cortex | 0.63 |
| 65 | 60.2 | -22.1 | 52.6 | 1 | Primary Somatosensory Cortex | 0.25 |
| | | | | 2 | Primary Somatosensory Cortex | 0.30 |
| | | | | 3 | Primary Somatosensory Cortex | 0.22 |
| | | | | 4 | Primary Motor Cortex | 0.07 |
| | | | | 6 | Pre Motor and Supplementary Motor Cortex | 0.09 |
| | | | | 40 | Supramarginal gyrus part of Wernicke's area | 0.08 |
| 66 | 69.2 | -24.2 | 32.3 | 1 | Primary Somatosensory Cortex | 0.21 |
| | | | | 2 | Primary Somatosensory Cortex | 0.24 |
| | | | | 3 | Primary Somatosensory Cortex | 0.10 |
| | | | | 4 | Primary Motor Cortex | 0.00 |
| | | | | 6 | Pre Motor and Supplementary Motor Cortex | 0.01 |
| | | | | 40 | Supramarginal gyrus part of Wernicke's area | 0.45 |
| 67 | 72.4 | -25.8 | 2.8 | 21 | Middle Temporal gyrus | 0.26 |
| | | | | 22 | Superior Temporal Gyrus | 0.48 |
| | | | | 42 | Primary and Auditory Association Cortex | 0.26 |
| 68 | -69.3 | -30.7 | 15.2 | 22 | Superior Temporal Gyrus | 0.43 |
| | | | | 40 | Supramarginal gyrus part of Wernicke's area | 0.17 |
| | | | | 42 | Primary and Auditory Association Cortex | 0.40 |
| 69 | -65.1 | -27.4 | 40.2 | 1 | Primary Somatosensory Cortex | 0.19 |
| | | | | 2 | Primary Somatosensory Cortex | 0.23 |
| | | | | 3 | Primary Somatosensory Cortex | 0.10 |
| | | | | 4 | Primary Motor Cortex | 0.03 |
| | | | | 6 | Pre Motor and Supplementary Motor Cortex | 0.01 |
| | | | | 40 | Supramarginal gyrus part of Wernicke's area | 0.44 |
| 70 | -53.5 | -22.9 | 58.3 | 1 | Primary Somatosensory Cortex | 0.30 |
| | | | | 2 | Primary Somatosensory Cortex | 0.35 |
| | | | | 3 | Primary Somatosensory Cortex | 0.29 |
| | | | | 4 | Primary Motor Cortex | 0.00 |
| | | | | 40 | Supramarginal gyrus part of Wernicke's area | 0.06 |

| | | | | | | |
|---|---|---|---|---|---|---|
| 71 | -35.9 | -20.1 | 71.7 | 3 | Primary Somatosensory Cortex | 0.12 |
| | | | | 4 | Primary Motor Cortex | 0.20 |
| | | | | 6 | Pre Motor and Supplementary Motor Cortex | 0.68 |
| 72 | -15.2 | -20.2 | 78.2 | 4 | Primary Motor Cortex | 0.21 |
| | | | | 6 | Pre Motor and Supplementary Motor Cortex | 0.79 |
| 73 | 11.4 | -22.9 | 78.7 | 4 | Primary Motor Cortex | 0.15 |
| | | | | 6 | Pre Motor and Supplementary Motor Cortex | 0.85 |
| 74 | 32.3 | -25.1 | 73.7 | 3 | Primary Somatosensory Cortex | 0.15 |
| | | | | 4 | Primary Motor Cortex | 0.42 |
| | | | | 6 | Pre Motor and Supplementary Motor Cortex | 0.43 |
| 75 | 52.2 | -30.5 | 61.2 | 1 | Primary Somatosensory Cortex | 0.26 |
| | | | | 2 | Primary Somatosensory Cortex | 0.24 |
| | | | | 3 | Primary Somatosensory Cortex | 0.14 |
| | | | | 40 | Supramarginal gyrus part of Wernicke's area | 0.36 |
| 76 | 65.8 | -36.3 | 42.9 | 1 | Primary Somatosensory Cortex | 0.03 |
| | | | | 2 | Primary Somatosensory Cortex | 0.10 |
| | | | | 40 | Supramarginal gyrus part of Wernicke's area | 0.87 |
| 77 | 70.6 | -38.1 | 17.0 | 22 | Superior Temporal Gyrus | 0.65 |
| | | | | 40 | Supramarginal gyrus part of Wernicke's area | 0.19 |
| | | | | 42 | Primary and Auditory Association Cortex | 0.15 |
| 78 | -68.8 | -42.8 | -2.5 | 21 | Middle Temporal gyrus | 0.77 |
| | | | | 22 | Superior Temporal Gyrus | 0.23 |
| 79 | -67.6 | -39.9 | 26.0 | 22 | Superior Temporal Gyrus | 0.23 |
| | | | | 40 | Supramarginal gyrus part of Wernicke's area | 0.76 |
| | | | | 42 | Primary and Auditory Association Cortex | 0.02 |
| 80 | -61.5 | -36.8 | 47.5 | 1 | Primary Somatosensory Cortex | 0.04 |
| | | | | 2 | Primary Somatosensory Cortex | 0.11 |

| | | | | | | |
|---|---|---|---|---|---|---|
| | | | | 40 | Supramarginal gyrus part of Wernicke's area | 0.85 |
| 81 | -46.8 | -31.4 | 64.7 | 1 | Primary Somatosensory Cortex | 0.28 |
| | | | | 2 | Primary Somatosensory Cortex | 0.27 |
| | | | | 3 | Primary Somatosensory Cortex | 0.25 |
| | | | | 40 | Supramarginal gyrus part of Wernicke's area | 0.19 |
| 82 | -25.3 | -33.1 | 75.1 | 1 | Primary Somatosensory Cortex | 0.06 |
| | | | | 2 | Primary Somatosensory Cortex | 0.17 |
| | | | | 3 | Primary Somatosensory Cortex | 0.35 |
| | | | | 4 | Primary Motor Cortex | 0.39 |
| | | | | 5 | Somatosensory Association Cortex | 0.04 |
| 83 | -2.9 | -35.5 | 78.5 | 3 | Primary Somatosensory Cortex | 0.10 |
| | | | | 4 | Primary Motor Cortex | 0.34 |
| | | | | 5 | Somatosensory Association Cortex | 0.05 |
| | | | | 6 | Pre Motor and Supplementary Motor Cortex | 0.52 |
| 84 | 21.2 | -37.4 | 77.2 | 2 | Primary Somatosensory Cortex | 0.11 |
| | | | | 3 | Primary Somatosensory Cortex | 0.46 |
| | | | | 4 | Primary Motor Cortex | 0.27 |
| | | | | 5 | Somatosensory Association Cortex | 0.16 |
| 85 | 42.8 | -40.5 | 66.0 | 1 | Primary Somatosensory Cortex | 0.13 |
| | | | | 2 | Primary Somatosensory Cortex | 0.31 |
| | | | | 3 | Primary Somatosensory Cortex | 0.08 |
| | | | | 4 | Primary Motor Cortex | 0.00 |
| | | | | 5 | Somatosensory Association Cortex | 0.28 |
| | | | | 40 | Supramarginal gyrus part of Wernicke's area | 0.20 |
| 86 | 58.9 | -47.5 | 48.7 | 40 | Supramarginal gyrus part of Wernicke's area | 1.00 |
| 87 | 66.1 | -50.1 | 25.8 | 22 | Superior Temporal Gyrus | 0.26 |
| | | | | 39 | Angular gyrus, part of Wernicke's area | 0.06 |
| | | | | 40 | Supramarginal gyrus part of Wernicke's area | 0.68 |
| 88 | 67.4 | -51.4 | -2.1 | 21 | Middle Temporal gyrus | 0.62 |

| | | | | | | |
|---|---|---|---|---|---|---|
| | | | | 22 | Superior Temporal Gyrus | 0.07 |
| | | | | 37 | Fusiform gyrus | 0.31 |
| 89 | -66.5 | -52.1 | 7.9 | 21 | Middle Temporal gyrus | 0.47 |
| | | | | 22 | Superior Temporal Gyrus | 0.53 |
| 90 | -63.7 | -49.4 | 34.2 | 39 | Angular gyrus, part of Wernicke's area | 0.02 |
| | | | | 40 | Supramarginal gyrus part of Wernicke's area | 0.98 |
| 91 | -54.5 | -46.4 | 53.6 | 40 | Supramarginal gyrus part of Wernicke's area | 1.00 |
| 92 | -37.0 | -45.6 | 68.6 | 1 | Primary Somatosensory Cortex | 0.03 |
| | | | | 2 | Primary Somatosensory Cortex | 0.27 |
| | | | | 5 | Somatosensory Association Cortex | 0.49 |
| | | | | 7 | Somatosensory Association Cortex | 0.12 |
| | | | | 40 | Supramarginal gyrus part of Wernicke's area | 0.09 |
| 93 | -15.7 | -47.2 | 76.8 | 2 | Primary Somatosensory Cortex | 0.03 |
| | | | | 3 | Primary Somatosensory Cortex | 0.21 |
| | | | | 4 | Primary Motor Cortex | 0.04 |
| | | | | 5 | Somatosensory Association Cortex | 0.38 |
| | | | | 7 | Somatosensory Association Cortex | 0.34 |
| 94 | 11.5 | -48.8 | 76.6 | 3 | Primary Somatosensory Cortex | 0.09 |
| | | | | 4 | Primary Motor Cortex | 0.10 |
| | | | | 5 | Somatosensory Association Cortex | 0.39 |
| | | | | 7 | Somatosensory Association Cortex | 0.41 |
| 95 | 31.5 | -53.5 | 69.4 | 5 | Somatosensory Association Cortex | 0.30 |
| | | | | 7 | Somatosensory Association Cortex | 0.70 |
| 96 | 48.6 | -58.1 | 53.5 | 7 | Somatosensory Association Cortex | 0.23 |
| | | | | 40 | Supramarginal gyrus part of Wernicke's area | 0.78 |
| 97 | 58.2 | -62.3 | 32.8 | 39 | Angular gyrus, part of Wernicke's area | 0.64 |
| | | | | 40 | Supramarginal gyrus part of Wernicke's area | 0.36 |

| | | | | | | |
|---|---|---|---|---|---|---|
| 98 | 61.4 | -63.7 | 6.8 | 19 | V3 | 0.13 |
| | | | | 21 | Middle Temporal gyrus | 0.27 |
| | | | | 22 | Superior Temporal Gyrus | 0.12 |
| | | | | 37 | Fusiform gyrus | 0.30 |
| | | | | 39 | Angular gyrus, part of Wernicke's area | 0.19 |

*Coordinates are based on the MNI system and (-) on the x-axis indicates left hemisphere.

**BA: Brodmann's Area. MNI coordinates were converted to Talairach coordinates to generate cluster labels.
